# Phenotype and Genotype Interaction Underlying Distributive Characteristic for Awn Development in Rice

**DOI:** 10.3390/plants11070851

**Published:** 2022-03-23

**Authors:** Jae-Ryoung Park, Ju Hyeong Son, Eun-Gyeong Kim, Yoon-Hee Jang, Byoung-Ju Yun, Kyung-Min Kim

**Affiliations:** 1 Crop Breeding Division, National Institute of Crop Science, Rural Development Administration, Wanju 55365, Korea; icd0192@korea.kr; 2 Division of Plant Biosciences, School of Applied Biosciences, College of Agriculture and Life Sciences, Kyungpook National University, Daegu 41566, Korea; ff0319@hanmail.net (J.H.S.); dkqkxk632@naver.com (E.-G.K.); uniunnie@naver.com (Y.-H.J.); 3 School of Electronic and Electrical Engineering, College of IT Engineering, Kyungpook National University, 80, Daehak-ro, Buk-gu, Daegu 41566, Korea; 4 Coastal Agriculture Research Institute, Kyungpook National University, Daegu 41566, Korea

**Keywords:** awn, rice, breeding, QTL, agricultural

## Abstract

As a wild ancestor of cultivated rice, *Oryza rufipogon* is domesticated into cultivated rice *Oryza sativa*, many agricultural traits are newly created or disappear. In particular, in wild rice, awn protects from predators and is easily blown by the wind and used as a means of propagation. However, awns gradually disappeared as they were breeding from wild rice to cultivated rice. Since awn development is disadvantageous to rice yield, it is important to understand the genetic basis related to awn development. In addition, characterization of the genes associated with awn development is helpful in analyzing the genetic relationships of rice from ancient times to the present for the regulatory mechanisms of awn formation. QTL analysis identified RM14330-RM218 on chromosome 3 using a 120 Cheongcheong/Nagdong double haploid population. Through screening of genes related to awn development in RM-14330-RM218, it is indicated that *OsDRPq3* is a causal gene that can be involved in awn development. *OsDRPq3* transcription level is maintained high in long awn and less yield populations during the panicle formation stage, the period during awn development. Moreover, the sequence of *OsDRPq3* has high homology with the drooping protein leaf. This study provides a new resource for phylogenetic research of rice and exploration of awn development.

## 1. Introduction

*Oryza sativa* was domesticated from wild rice *Oryza rufipogon* in Asia about 8000 years ago [1]. New traits emerged during breeding by humans, and as various traits disappeared, many traits of *O. rufipogon* changed considerably [2]. The ancient *O. rufipogon* was aimed at maintaining offspring and breeding. However, cultivated rice *O. sativa* was selected and bred to obtain more convenience, preferable taste, and yield for agricultural activities [3]. Representatively, the presence of awn can protect rice from predators, and awn is easily blown by the wind or attached to the body of an animal, making it a useful means for breeding species [4,5]. However, in modern times, the presence of awn causes malfunction when operating agricultural machinery, and a missing plant easily occurs. In addition to awn, shattering, viviparousness, and after-wintering seed viability are the main agricultural characteristics not found in cultivated rice but only in wild rice, and the characteristics that distinguish wild rice from cultivated rice are called domestication traits [6]. Therefore, these agricultural characteristics are also major factors that can distinguish between cultivated rice and wild rice.

Awn is one of the important morphologically distinct characteristics of rice seeds [7] and is also found in rice, barley, oats, wheat, and sorghum belonging to Gramineae. The awn of rice is removed during domestication. However, in wheat and barley, awn continues to enable photosynthesis, and the photosynthetic products produced by awn increase yields [8]. Cultivated rice is subjected to breeding to remove awn or maintain a very short length because awn does not contribute significantly to photosynthesis [9]. The removal of rice awn does not negatively affect rice grain maturation, so only awn is removed for the convenience of harvesting and storage [10]. The presence or absence of rice awn varies depending on the cultivar, and the length of the awn formed from a single panicle is very variable, even in plants with awn. Therefore, genes have very complex interactions in the formation of awn development [11,12,13].

Genetic analysis shows that awns are quantitative trait loci (QTL) involving polygene, and awn length and development-related QTL have been reported in segregating populations developed through crossing between wild rice and cultivated rice [14,15]. Since the development of QTL analyses, many genes have been completely cloned; recently, it has been known that An-1 encoding the helix-loop-helix protein is cloned and involved in awn elongation [16]. However, molecular mechanisms involved in awn development in rice have not been fully elucidated.

Awn is one of the very interesting traits in rice breeding. In particular, it is an element that can be effectively used in the phylogenetics of rice ancestors because it is a characteristic that has been removed from ancient rice through domestication in modern times [17,18]. Long awns are easily blown by the wind and are an effective means of breeding seeds. However, awn has become a trait that could not be selected due to domestication. In particular, the development of awn decreases the grain number per plant [16]. This study identified loci related to awn and yield and demonstrated that awn elongation negatively affects yield. Associate genes affecting awn length in the QTL region were screened. The relative expression level of the associate genes screened in the awned and awnless populations was analyzed, and the results can be effectively used to create a phylogenetic tree of awned rice and develop awnless rice cultivars with a large quantity.

## 2. Results

### 2.1. Phenotype Characterization Associated with Awn Elongation in 120 Cheongcheong/Nagdong Double Haploid (CNDH) Population

**Figure 1:**
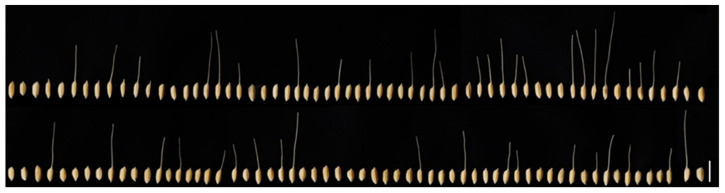
Phenotype of various awn lengths in the CNDH population. *O. sativa* spp. *indica* cv. Cheongcheong and *O. sativa* spp. *japonica* cv. Nagdong were crossed to obtain F_1_. F_1_ was another cultured to create a 120 CNDH population. Due to the *indica* type, Cheongcheong, the presence or absence of awn in the 120 CNDH population was very diverse. Bars, 10 mm.

**Figure 2:**
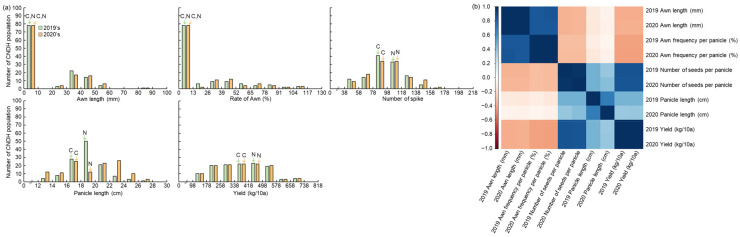
Frequency distribution and heat map for agricultural traits. (**a**) Frequency distribution for awn development traits in the CNDH population. All agricultural traits, such as awn length, awn frequency per panicle, number of seeds per panicle, panicle length, and yield, of the CNDH population were of normal distribution. Therefore, awn development is regulated by quantitative traits. C, Cheongcheong; N, Nagdong. (**b**) Heat map showing the correlation matrix for agricultural traits related to awn development. The color corresponds to the strength of correlations (blue, positive correlation; white, no correlation; red, negative correlation). Awn length and awn frequency per panicle have a very high level of positive correlation, and awn length and yield have a very high level of negative correlation.

To analyze QTLs related to awn elongation and yield, the panicle length, number of seeds per panicle, awn length, awn frequency per panicle, and yield of the Cheongcheong, Nagdong, and 120 CNDH populations were investigated in 2019 and 2020 (Figure 1 and Table S1). In Cheongcheong and Nagdong, awns were not developed, and awns were developed only in the CNDH population. The number of seeds per panicle was highest in Nagdong and lowest in the DH population. In addition, panicle length was longest in Nagdong and shortest in the DH population. In addition, the yield was the most in Nagdong and the lowest in Cheongcheong. This trend has been maintained for two consecutive years. The Cheongcheong, Nagdong, and 120 CNDH populations’ panicle length, number of seeds per panicle, awn length, awn frequency per panicle, and yield measurements using histogram analysis showed a continuous variation close to the normal distribution (Figure 2a). These agricultural traits are quantitative traits in which polygenes are involved. In addition, when the correlation between these various agricultural traits was analyzed, awn length and awn frequency per panicle (2019 *r* = 0.846, 2020 *r* = 0.850) were positively correlated, and awn length, number of spikelets (2019 *r* = −0.346, 2020 *r* = −0.331), panicle length (2019 *r* = −0.112, 2020 *r* = −0.067), and yield (2019 *r* = −0.364, 2020 *r* = −0.364) were negatively correlated with each other. Among these, awn length and yield have a very high negative correlation (Figure 2b and Table S2).

### 2.2. Awn Elongation and Yield Based on QTL Mapping and Physical Map Construct

**Figure 3:**
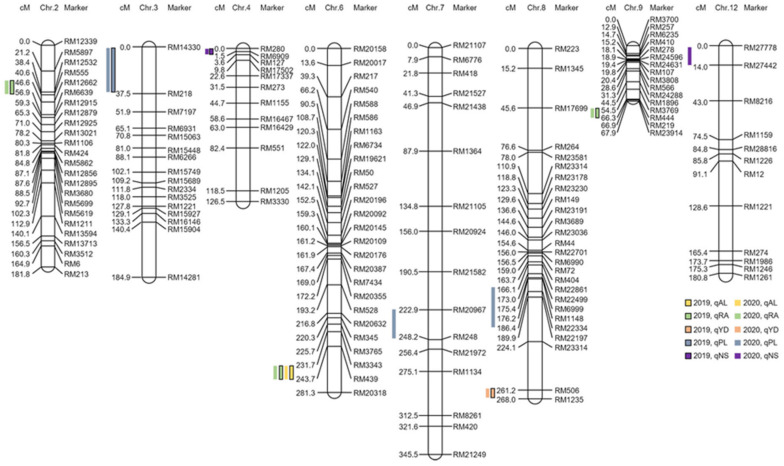
Chromosomal location of the QTLs associated with awn development. QTL mapping results indicate that awn development and elongation-related genes are located on chromosomes 2 to 4, 6 to 9, and 12. Among the detected QTLs, RM12662-RM6639 on chromosome 2, RM14330-RM218 on chromosome 3, RM280-RM6909 on chromosome 4, RM3343-RM439 on chromosome 6, RM506-RM1235 on chromosome 8, and RM3769-RM444 on chromosome 9 were detected in regions with an LOD score of 3.0 or higher for two consecutive years.

To construct a genetic map of the CNDH population, SSR markers with polymorphisms were selected in Cheongcheong and Nagdong. Polymorphism was analyzed for 788 SSR markers in Cheongcheong and Nagdong, and among them, 222 SSR markers with polymorphism confirmed in Cheongcheong and Nagdong were selected for the genetic map construct. The constructed CNDH genetic map has 19 to 50 markers per chromosome. The 222 SSR markers used in 12 chromosomes are evenly distributed, and a genetic map with a distance between markers of 10.6 cm and a total length of 2121.7 cm was finally constructed (Figure 3). QTL mapping was performed using the panicle length, number of seeds per panicle, awn length, awn frequency per panicle, and yield data of the 120 CNDH population. In the QTL mapping analysis, *qRA2* on chromosome 2, *qPL3* on chromosome 3, *qNS4* on chromosome 4, *qAL6* and *qRA6* on chromosome 6, *qPL7* on chromosome 7, and *qPL8* and *qYD8* on chromosome 8 were detected in 2019 and *qRA2-1* on chromosome 2, *qPL3-1* on chromosome 3, *qNS4-1* on chromosome 4, *qAL6-1* and *qRA6-1* on chromosome 6, *qYD8-1* on chromosome 8, *qRA9-1* on chromosome 9, and *qNS12* on chromosome 12 were detected in 2020 (Table S3). *qRA2*, *qPL3*, *qNS4*, *qAL6*, *qRA6*, *qPL7*, *qPL8*, *qYD8*, and *qRA9* were detected in 2019, and *qRA2-1*, *qPL3-1*, *qNS4-1*, *qAL6-1*, *qRA6-1*, *qYD8-1*, *qRA9-1*, and *qNS12* were detected in 2020. *qAL6* and *qAL6-1* were QTLs related to awn length and detected in 2019 and 2020, respectively. *qAL6* was located at RM3343-RM439 on chromosome 6, the logarithm of the odds (LOD) score was 3.3, and the explainable phenotypic variation was 10%, derived from the Cheongcheong allele. *qAL6-1* was located at RM3343-RM439 on chromosome 6, the LOD score was 3.0, and the descriptive phenotypic variation was 10%, derived from the Cheongcheong allele. For the panicle length-related QTLs, *qPL3*, *qPL7*, and *qPL8* were detected in 2019, and *qPL3-1* was detected in 2020. *qPL3* was located at RM14330-RM218 on chromosome 3, and the LOD score was 3.2, derived from the Cheongcheong allele. *qPL7* was located at RM20967-RM248 on chromosome 7, the LOD score was 2.9, and the explainable phenotypic variation was 10%, derived from the Cheongcheong allele. *qPL8* was located at RM22861-RM22334 on chromosome 8, the LOD score was 5.1, and the explainable phenotypic variation was 10%, derived from the Nagdong allele. *qPL3-1* was located at RM14330-RM218 on chromosome 3, the LOD score was 3.6, and the descriptive phenotypic variation was 20%, derived from the Cheongcheong allele. The QTLs related to the number of spikelets were searched for *qNS4* in 2019 and *qNS4-1* and *qNS12* in 2020. *qNS4* was located at RM280-RM6909 on chromosome 4, the LOD score was 2.7, and the explainable phenotypic variation was 10%, derived from the Nagdong allele. *qNS4-1* was located at RM280-RM6909 on chromosome 4, the LOD score was 2.8, and the explainable phenotypic variation was 10%, derived from the Nagdong allele. *qNS12* was located at RM27778-RM27442 on chromosome 12, the LOD score was 2.7, and the explained phenotypic variation was 20%, derived from the Nagdong allele. QTLs related to the rate of awn for *qRA2*, *qRA6*, and *qRA9* were detected in 2019 and *qRA2-1*, *qRA6-1*, and *qRA9-1* in 2020. *qRA2* was located at RM12662-RM6639 on chromosome 2, the LOD score was 4.8, and the explainable phenotypic variation was 10%, derived from the Cheongcheong allele. *qRA6* was located at RM3343-RM439 on chromosome 6, the LOD score of 3.5, and the explainable phenotypic variation was 10%, derived from the Cheongcheong allele. *qRA9* was located at RM3769-RM444 on chromosome 9, the LOD score was 3.3, and explainable phenotypic variation was 10%, derived from the Cheongcheong allele. *qRA2-1* was located at RM12662-RM6639 on chromosome 2, the LOD score was 4.6, and the explainable phenotypic variation was 10%, derived from the Nagdong allele. *qRA6-1* was located at RM3343-RM439 on chromosome 6, the LOD score was 3.5, and the explainable phenotypic variation was 10%, derived from the Cheongcheong allele. *qRA9-1* was located at RM3769-RM444 on chromosome 9, the LOD score was 3.4, and the explainable phenotypic variation was 10%, derived from the Cheongcheong allele. For yield-related QTLs, *qYD8* was detected in 2019 and qYD8-1 in 2020. *qYD8* was located at RM506-RM1235 on chromosome 8, the LOD score was 3.1, and the explainable phenotypic variation was 10%, derived from the Cheongcheong allele. *qYD8-1* was located at RM605-RM1235 on chromosome 8, the LOD score was 3.1, and the explainable phenotypic variation was 10%, derived from the Cheongcheong allele.

### 2.3. Search for Associate Genes Associated with Awn Development Based on QTL Mapping

**Figure 4:**
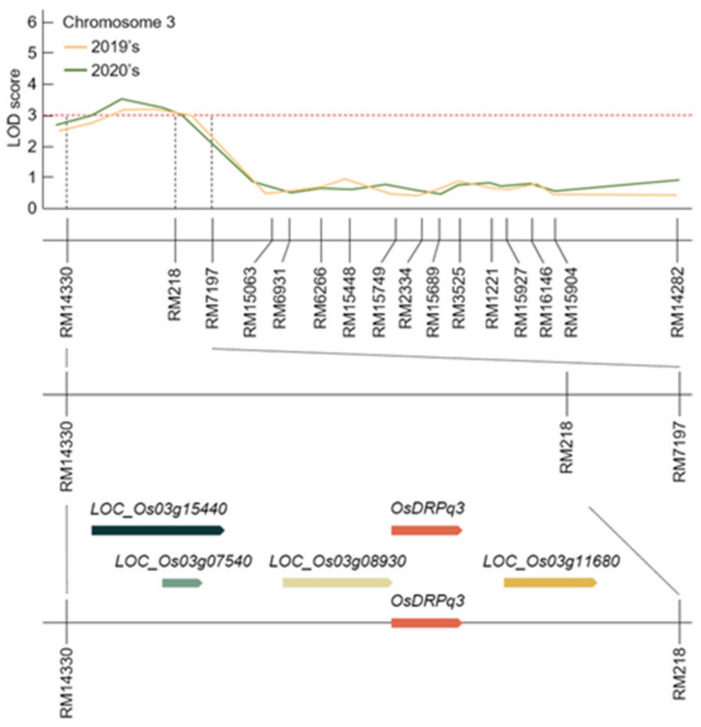
Physical map related to awn development and yield genes. The LOD score of RM14330-RM197 in chromosome 3 was 3.0, and the LOD score of RM14330-RM197 was 3.6. In particular, RM14330-RM218 was commonly detected with an LOD score of 3.0 or higher, and screening of all ORFs detected was related to awn development. LOC_Os03g15440, LOC_Os03g07540, and LOC_Os03g08930 are related to bHLH dimerization region bHLH domain-containing protein. LOC_Os03g11600 is related to drooping leaf protein. LOC_Os03g11680 is related to similar to zinc finger protein. LOC_Os03g11600 is a gene involved in yield and awn elongation. Therefore, LOC_Os03g11600 was finally detected among five ORFs and named *OsDRPq3*.

Using the investigated characteristics, QTL mapping was analyzed to screen open reading frames (ORFs) related to awn elongation in the detected region. The detected QTL region had 22.2% helix-loop-helix DNA-binding domain region proteins, 44.4% basic helix-loop-helix (bHLH) dimerization region proteins, 14.8% winged-helix repressor DNA-binding domain proteins, and 3.7% each of drooping leaf protein, similar to zinc finger protein, epidermal growth factor receptor protein, filamentous flower protein, and auxin-responsive protein (Table S4). Among these ORFs, those with a coding sequence (CDS) size of 400 to 800 bp were selected (Figure 4). Among the screened ORFs, *LOC_Os02g08220* and *LOC_Os08g16030* contained helix-loop-helix DNA-binding domain region, and *LOC_Os08g37290*, *LOC_Os04g52770*, and *LOC_Os07g08440* contained bHLH dimerization region. *LOC_Os12g10140* codes for winged-helix repressor DNA-binding domain, *LOC_Os03g11600* (*OsDRPq3*) codes for drooping leaf protein, and *LOC_Os07g06620* codes for filamentous flower protein, and these genes were selected as associate genes to awn elongation. In particular, *OsDRPq3* was located at RM14330-RM218 on chromosome 3. In 2019, RM14330-RM7197 was detected with an LOD score of 3.2; in 2020, RM1430-RM218 was detected with an LOD score of 3.6. Therefore, this study focused on RM14330-RM218, a region commonly detected for 2 years. At RM14330-RM218, ORFs related to awn elongation, *LOC_Os03g15440*, *LOC_Os03g07540*, *LOC_Os03g08930*, *OsDRPq3*, and *LOC_Os03g11680*, were screened. Among these, *LOC_Os03g15440*, *LOC_Os03g07540*, and *LOC_Os03g08930* are proteins including the bHLH dimerization region, and the length of each CDS region was 1230, 276, and 990 bp, respectively. *OsDRPq3* is a drooping leaf protein, and the CDS size is 591 bp. *LOC_Os03g11680* is similar to zinc finger protein, and the CDS size is 894 bp.

### 2.4. Associate Gene Expression Level at the Panicle Formation Stage

**Figure 5:**
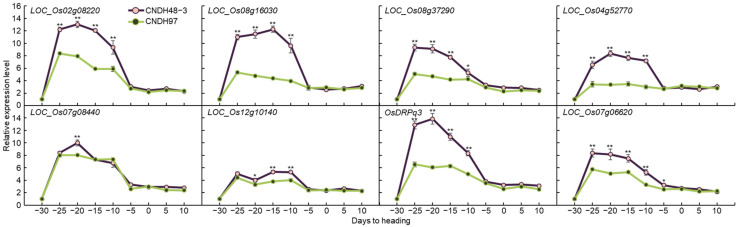
Comparison of relative expression levels of associate genes related to awn development and yield according to the development stage for awn. The associate genes detected by QTL mapping to the relative expression levels were compared. CNDH48-3 is a population with a long awn and low yield. CNDH97 is a population with a high yield and without awn elongation. All associate genes were expressed at higher levels in CNDH48-3 than in CNDH97. However, *OsDRPq3* had the most difference in expression levels between CNDH48-3 and CNDH97. Therefore, *OsDRPq3* was finally selected. * Significant at the 0.05 level; ** significant at the 0.01 level.

The relative expression levels of associate genes to awn elongation were analyzed at the panicle formation stage. To analyze the change in the relative expression level during the panicle formation stage when the awn of the associate genes began to develop, the panicles were sampled at intervals of 5 to 30 days before heading to 10 days after heading and sampled nine times (Figure 5). In the 120 CNDH population, CNDH48-3 has a long awn length and low yield, and CNDH97 is an awnless line with a large yield. *LOC_Os02g08220*, *LOC_Os08g16030*, *LOC_Os04g52770*, and *OsDRPq3* had a significant difference at the 1% level in CNDH48-3 compared to CNDH97 at 25, 20, 15, and 10 days before heading. *LOC_Os08g37290* had a higher relative expression level of CNDH48-3 at the 5% level at 10 days before heading. At the 1% level at 25, 30, and 15 days before heading, the relative expression level was higher in CNDH48-3 than CNDH97. *LOC_Os07g08440* had a high relative expression level of CNDH48-3 at the 1% level at 20 days before heading. *LOC_Os12g10140* had a high relative expression level of CNDH48-3 at the 5% level at 20 days before heading. At the 1% level at 15 and 10 days before heading, the relative expression level was higher in CNDH48-3 than CNDH97. *LOC_Os07g06620* had a higher relative expression level of CNDH48-3 at the 1% level at 25, 20, 15, and 10 days before heading and a higher relative expression level of CNDH48-3 at the 5% level at 5 days before heading.

### 2.5. Analysis of the Phylogenetic Tree and Homology Sequence

**Figure 6:**
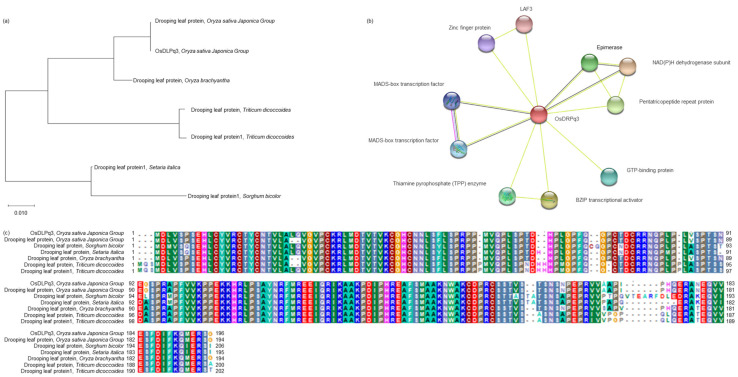
Homology analysis of *OsDRPq3*. (**a**) *OsDRPq3* has very high homology to genes related to awn elongation in wheat, barley, and sorghum belonging to Gramineae. (**b**) *OsDRPq3* was analyzed with very high homology with drooping leaf protein of crops belonging to Gramineae. (**c**) Especially when predicting protein interaction, *OsDRPq3* is associated with genes related to signaling and awn development. *OsDRPq3* can be effectively used to breed high yield rice without awn in the future.

Among the associated genes to awn elongation, *OsDRPq3* codes for drooping leaf protein. Using the *OsDRPq3* sequence, the genetic similarity to drooping leaf protein in *O. sativa*, *Oryza brachyantha*, *Triticum dicoccoides*, *Sorghum bicolor*, and *Setaria italica* belonging to Gramineae was analyzed using a phylogenetic tree (Figure 6a). *OsDRPq3* belonged to the same group as the drooping leaf protein of *Oryza*, and the genetic similarity to the drooping leaf protein of *O. sativa* was 99% identical (194/196) and similarity (98%). In addition, the genetic similarity to drooping leaf protein 1 of *S. bicolor* was 88% identical (176/200) and 90% similar (Figure 6b). When predicting the functional proteins using the *OsDRPq3* domain, 10 different proteins (LAF3, zinc finger protein, MADS-box transcription factor 1 and 2, thiamine pyrophosphate (TPP) enzyme, BZIP transcriptional activator, GTP-binding protein, pentatricopeptide repeat protein, NADPH dehydrogenase subunit, and RNA-binding protein) interacted with *OsDRPq3* (Figure 6c).

## 3. Discussion

Awn is one of the important agricultural traits that disappeared during the domestication of wild rice, such as *O. rufipogon* or *O. barthii*, into cultivated rice such as *O. sativa* or *O. glaberrima* [19]. Therefore, awn is an element that can be effectively used to distinguish rice ancestors. The awn is also an element that disappeared during domestication to effectively handle harvesting and postharvest processing. In this study, the *indica* type, *O. sativa* cv. Cheongcheong and *japonica*-type *O. sativa* cv. Nagdong was crossed to create F_1_. F_1_ was anther cultured, and the constructed 120 CNDH population was used. Cheongcheong and Nagdong are awnless; because Cheongcheong is an *indica* type, both lines with awn and lines without awn were present in the 120 CNDH population [20]. Cheongcheong and Nagdong are rice cultivars cultivated for a long time in Korea, and both of awnless cultivars. However, both awn-developed and awnless populations exist in the CNDH populations derived through the cross of Cheongcheong and Nagdong because there is a cultivar with awn developed in the ancestors of Cheongcheong and Nagdong [21]. Cheongcheong and Nagdong are awnless phenotypic but genetically have awn loci, and these genes are not expressed by other genes. Therefore, awn is one of the wild-specific rice traits, and if a locus related to awn is found, it can be effectively used to create a genealogy of rice from the ancestors to the present [22].

In this study, QTL mapping was analyzed using 120 CNDH populations to detect loci that play a key role in awn elongation and development. Awn length and the rate of awn are close to a qualitative trait, but the number of spikelets, panicle length, and yield follow a normal distribution and are quantitative traits with a continuous variation. Quantitative traits 261 are phenotypes of traits encoded not only by one gene but also by the interaction of various genes [23,24]. In particular, awn has a very strong negative correlation with the number of spikelets, panicle length, and yield that directly affects quantity [25]. As wild rice became cultivated rice through domestication, major agricultural traits were changed [26].

Wild rice has long developed awns and very easy shattering. The lifespan of pollen is long, and the size of seeds is small. The number of seeds formed on each panicle is small, and the panicle shape is small and scattered. However, as wild rice is domesticated, the awn is very short or absent, and shattering is difficult. In addition, the size of seeds is large, many seeds form on the panicle, and the ear shape is long and dense. All these phenotypic differences are related to yield, and wild rice has morphological characteristics that are very advantageous for seed propagation. However, cultivated rice was bred to increase the yield to solve the food problem [27].

Through QTL mapping for 2 years, 17 QTL regions with an LOD score of 2.5 or higher were detected in eight chromosomes. Among these QTLs, *qPL3* and *qPL3-1* were detected with an LOD score of 3.0 or higher for two consecutive years at RM14330-RM218 on chromosome 3. In addition, both *qPL3* and *qPL3-1* were derived from the Cheongcheong allele, an *indica*. *Indica* is still mostly phenotypic of wild rice traits. To screen associate genes to awn development and elongation, ORFs present at RM14330-RM218 were analyzed, and *OsDRPq3* was selected among several ORFs. The awn development-related candidates in the QTL region and the relative expression level of *OsDRPq3* were analyzed during the panicle formation stage. The panicle formation stage is the time when factors related to awn development and quantity, such as ear shape, ear count, ear size, and awn development, are determined. Therefore, in this study, the panicle formation stage was sampled every 5 to 30 days before heading to 10 days after heading to analyze the relative expression level of associate genes. *OsDRPq3* had a significant difference in the relative expression level at the 1% level in CNDH48-3 than in CNDH97 at 25, 20, 15, and 10 days before heading. *OsDRPq3* was very similar in sequence to drooping leaf protein present in *O. brachyantha*, *T. dicoccoides*, *S. italica*, and *S. bicolor*. *O. brachyantha*, *T. dicoccoides*, *S. italica*, and *S. bicolor* are plants in which awn is developed, and drooping leaf protein plays a key role in awn development [28]. *OsDRPq3* interacts with LAF3, zinc finger protein, MADS-box transcription factor 1 and 2, TPP enzyme, BZIP transcriptional activator, GTP-binding protein, pentatricopeptide repeat protein, NADPH dehydrogenase subunit, and epimerase. Among these proteins, MADS-box, TPP enzyme, and BZIP are important transcription factors in plants. RNA-binding protein, NAD(P)H dehydrogenase subunit, and pentatricopeptide repeat protein act on signaling and morphogenesis in plants through RNA modulating. In particular, RNA processing mediates the growth and development of auxin and cytokinin, auxin, and cytokinin [29]. An-2 catalyzes the reaction of the last stage of cytokinin synthesis and plays a key role in the awn elongation of *O. rufipogon* [30]. Zinc finger protein is involved in various regulatory processes in plants and also acts on cytokinin synthesis [31]. In addition, zinc finger protein is a highly conserved region only in plants, is expressed in most tissues, and can activate or repress transcription [5]. MADS-box transcription factors 1 and 2, TPP enzyme, and BZIP transcriptional activator are transcription factors that interact with OsDRPq3. MADS-box transcription factors are important regulators involved in all developmental processes of plants and are particularly involved in root and lateral elongation [32]. In rice, the SHI-family transcription factor is involved in pistil morphology and awn elongation. In addition to *Oryza*, *Arabidopsis* also shows a partial redundancy function as a conserved domain [33]. Drooping leaf protein is latent in *japonica* but is involved in awn elongation and development in *indica* and regulates awn development in a non-cell-autonomous manner [34]. GTP-binding protein is also responsible for signaling in plants and is involved in awn development and elongation [35,36]. OsDRPq3 is predicted to be involved in awn development and elongation by complexly interacting with each other with the aforementioned transcription factors, RNA processing, and GTP-binding protein. In this study, RM14330-RM218 on chromosome 3 detected for two consecutive years was mainly searched for genes related to awn development. DNA-binding domain-containing protein was detected, and bHLH dimerization region bHLH domain-containing protein was located at RM22861-RM22334 on chromosome 8. Awn-1 encodes a bHLH protein and is reported to regulate the long awn development of wild rice. Especially when Awn-1 is knocked down through RNA interference, the awn length is short. As a result, the number of grains per panicle decreased, resulting in a decrease in yield [30].

In addition, when QTL mapping was performed with the yield data of 120 CNDH population, the winged-helix repressor DNA-binding domain-containing protein was located at RM506-RM1235 on chromosome 8. These associate genes are involved in awn development and elongation; because the size of the gene is too large or too small, gene cloning is difficult, and if the size of the gene is large, it is difficult for complete genes to be transferred onto the next generation during meiosis. However, because associate genes involved in awn development were detected in the region detected through QTL mapping, the QTLs detected in this study can effectively create a breeding tree related to the evolution and development of awn in the future. In addition, Zhang et al. [37] analyzed QTL mapping related to awn length, which was detected in different chromosomes from this study. Although QTL mapping for the same trait was analyzed, the reason for mapping in different chromosomes is presumed to be because the groups used in the study were different, and the environmental differences and genetic factors of the groups were different [38]. In addition to the *OsDRPq3* screened in this study, the detected QTL region can be effectively used for awn development and awn genetic genealogy. In addition, rice lost awn during domestication and increased the grain number, ultimately increasing the yield. In this study, during domestication of rice, QTLs causing awn development gradually disappeared according to the breeder’s breeding goal and high yield, and as a result, traits that negatively affected yield were continuously reduced. Therefore, it is suggested that the traits were selected for harvest and storage and human preferences.

## 4. Materials and Methods

### 4.1. Plant Material and Field Design

The 120 CNDH population derived from the anther culture of F_1_ grown by crossing *O. sativa* spp. *indica* cv. Cheongcheong (IT228761, IT number is a resource number managed by the National Academy of Agricultural Sciences of Rural Development Administration, Korea) with *O. sativa* spp. *japonica* cv. Nagdong (IT006182) was used to identify loci that control awn elongation and yield. Prof. Kyung-Min Kim was provided Cheongcheong, Nagdong, and 120 CNDH population from the Plant Molecular Breeding laboratory (Kyungpook National University in the Republic of Korea). The 120 CNDH population was grown under natural conditions in the field of Kyungpook National University (36°6′41.54″ N, 128°38′26.17″ E) and sown and grown in a greenhouse before transplanting to the field. Seeds were sterilized using a seed disinfectant and soaked at 33 °C and in the dark for 3 days. Seeds were sown on 22 April 2019, and 20 April 2020, respectively, and after 30 days, in the field of Kyungpook National University. Thirty days after sowing, they were transplanted into the field of Kyungpook National University with a planting distance of 30 × 15 cm. The 120 CNDH population was bred by maintaining the standard fertilization amount N−P_2_O_5_−K_2_O = 9:4.5:5.7 kg/10 a according to the Agricultural Science and Technology Research Survey Standard of the RDA (Rural Development Administration). In addition, during the study, Cheongcheong, Nagdong, and 120 CNDH populations were used according to international guidelines and legislation provided by the RDA of Korea. All rice plants used in the study were grown according to normal local practices.

### 4.2. Measurement of Awn Related Phenotype and Yield

Various agriculture traits, such as panicle length, number of seeds per panicle, awn length, awn frequency per panicle, and yield, were measured for awn elongation and development-related QTL mapping. Panicle length was measured from the ear neck to the tip of the panicle. The number of seeds per panicle counted all spikelets in the panicle. Panicles that were completely emptied by compressing all spikelets in the ear were classified as sterile to determine the fertility rate. The awn length was measured using Vernier calipers, and those with an awn length longer than 3 mm were classified as awned. As for the awn length, the awn length of the whole panicle was investigated using the apical spikelet of the primary branch of each of the 120 CNDH populations. In addition, for each population, 25 main panicles were used. The awning frequency per panicle was measured among all spikelets in the panicle. For all characters examined, ten plants were repeated for each population.

### 4.3. Construction of Genetic Map and QTL Analysis Associated with Awn Elongation in Rice

The QTL mapping of genes affecting awn elongation and yield was analyzed using Window QTL cartographer 2.5. To construct a CNDH genetic map, polymorphisms were analyzed in Cheongcheong and Nagdong using the 788 SSR marker. Of these, 222 SSR markers that can distinguish between Cheongcheong and Nagdong were finally selected and used for genetic map construction, except for those that could not distinguish between Cheongcheong and Nagdong. Using 222 SSR markers, a genetic map with an average distance between markers of 10.6 cm was constructed using Mapmaker version 3.0 [39]. The physical positions of SSR markers were determined using Gramene (http://www.gramene.org, 16 November 2020). Composite Interval Mapping was used in the Kosambi Function of the Window QTL cartographer 2.5 for the awn elongation and yield of the 120 CNDH population, and the LOD score was 3.0 or higher to increase the accuracy of the detected QTLs. The naming of the detected QTLs followed the method proposed by McCouch [40].

### 4.4. Screening and Annotation of Associate Genes

After QTL mapping, associate genes related to awn elongation and yield were screened. Associate genes were selected using SSR markers in regions detected by QTL mapping using Rapdb (https://rapdb.dna.affrc.go.jp/ (accessed on 16 November 2020)) [41] and RiceXpro (https://ricexpro.dna.affrc.go.jp/ (accessed on 23 November 2020)) [42]. Rapdb and RiceXpro allow you to select ORFs for the detected QTL region. All ORFs in the QTL region were searched. In addition, these sites present all ORFs in the QTL region, classify ORFs according to function, and filter associate genes that affect awn elongation and yield. For sequence variation and homology analysis of screening associate genes, BioEdit 7.0 (http://www.mbio.ncsu.edu/BioEdit/BioEdit.html (accessed on 26 November 2020)) [43] and NCBI (http://www.ncbi.nim.nih.gov (accessed on 26 November 2020)) were used. In addition, a phylogenetic tree was constructed using MEGA 7.0 software (https://www.megasoftware.net/ (accessed on 28 November 2020)), with 1,000 bootstrap replicates based on amino acid sequence were applied. In addition, Simple Modular Architecture Research Tool or SMART (http://smart.embl-heidelberg.de/ (accessed on 28 November 2020)) [44] was used to predict their protein interaction.

### 4.5. Genomic DNA Extraction and PCR Protocol

NucleoSpin^®^ Plant II kit (Macherey-Nagel GmbH & Co., KG, Deutsch, Düren, Germany) was used to extract genomic DNA from a 120 CNDH population, Cheongcheong, and Nagdong. For DNA extraction, rice ears were made into powder using TissueLyser II (Qiagen, Hilden, Germany). PL2 buffer (300 µL) and RNase (10 µL) were added to 100 mg powder, mixed well, and reacted at 65 ℃ for 15 min. PL3 buffer (75 µL) was added and incubated at 4 ℃ for 5 min. After centrifugation at 13,000 rpm for 5 min, the mixture was transferred to a NucleoSpin filter (violet ring) and centrifuged at 13,000 rpm for 2 min. The filtered solution was transferred to a new 1.5 mL e-tube, and PC buffer (450 µL) was added and inverted. The sufficiently mixed solution was transferred to the NucleoSpin Plant II Column (green ring) and centrifuged for 1 min at 13,000 rpm. PW1 buffer (400 µL) was added and centrifuged at 13,000 rpm for 1 min. PW2 buffer (700 µL) was added and centrifuged at 13,000 rpm for 1 min. PW2 buffer (200 µL) was added and centrifuged at 13,000 rpm for 2 min. After each step, the filtered solution was discarded. The solution was transferred to the NucleoSpin Plant II Column (green ring) into a new 1.5 mL e-tube. PE buffer (50 µL) was added and incubated at 65 °C for 5 min. The solution was then centrifuged at 13,000 rpm for 2 min to dissolve the DNA from the column. A PCR mixture was prepared for PCR amplification of the candidate gene. The PCR mixture consisting of 100 ng template DNA, 10× Ex buffer (50 mM KCl, 20 mM Tris-HCl (pH 8.0), 2.0 mM MgCl_2_), 2.5 mM deoxynucleotriphosphates, 10 pmol forward primer, 10 pmol reverse primer, and 1 unit HS Prime Taq polymerase (cat. no. G-7002; Genet Bio, Daejeon, South Korea) was used, and a final volume of 50 µL was made using distilled water. The PCR (C1000; Bio-Rad, Hercules, CA, USA) conditions were pre-denaturation at 95 ℃ for 5 min, denaturation at 95 ℃ for 30 s, annealing at 55.5 ℃, extension at 72 ℃ for 1 min, and final extension at 72 ℃ for 5 min. Denaturation, annealing, and extension were performed 30 cycles so that the gene fragment can be sufficiently amplified, and when the reaction was completed, the mixture was stored at 4 ℃.

### 4.6. RNA Extraction

RNeasy plant mini kit (Qiagen, cat. 74904, Hilden, Germany) was used for RNA extraction of 120 CNDH population, Cheongcheong, and Nagdong, according to the manufacturer’s instructions. The panicle of rice was made into powder using a pestle bowl, and it was continuously frozen with liquid nitrogen to minimize the degeneration of RNA due to heat. Powder (80 mg) was added to a 2 mL e-tube, and RLT buffer (450 µL) was added. The mixed solution was transferred to a QIAshredder spin column and centrifuged for 5 min at 13,000 rpm. The filtered solution was transferred to a 1.5 mL e-tube, 1/2 volume of 100% ethanol was added, and the solution was mixed well and transferred to an RNeasy mini-column. After centrifugation at 13,000 rpm for 1 min, the filtrates were discarded. Then, RW1 buffer (700 µL) was added and centrifuged at 13,000 rpm, and the filtered solution was discarded. Then, RPE buffer (500 µL) was added and centrifuged at 13,000 rpm, and the filtered solution was discarded. The mixture was centrifuged once more at 13,000 rpm for 3 min without adding anything to completely dry the column filer. Then, to dissolve the RNA from the column, RNase-free water (40 µL) was added and centrifuged at 13,000 rpm for 1 min. The concentration and quality of the extracted RNA were analyzed using a Microvolume spectrophotometer (ND-2000; Thermo Scientific, Gangnam-gu, Seoul, Korea). RNA extracted after the analysis was diluted to 80 ng/µL using RNase-free water.

### 4.7. Analysis of the Associate Gene Expression Level

To find out whether the associate genes screened by QTL mapping affect awn elongation, panicles were collected at 5-day intervals for 30 days before heading and 10 days after heading, and the relative expression levels of associate genes involved in awn elongation were checked. Samples were rapidly cooled using liquid nitrogen and stored at −80 ℃ until they were used in experiments. RNA (2 μg) extracted using the RNeasy plant mini kit was used as a template for quantitative real-time PCR (qPCR). qPCR was analyzed using the Eco Real-time PCR System. For qPCR, 2× Real-time PCR Master Mix (10 µL BioFACT; cat. no. DQ384-40h; Gangnam-gu, Seoul, Korea), forward primer (10 pmol/µL; 1 µL), reverse primer (10 pmol/µL; 1 µL), 2 μg RNA, and double-distilled water were used to make a final volume of 20 μL (Table S5). The relative expression level of each associate gene was analyzed using *OsActin*, a housekeeping gene, as a control. Each reaction was carried out three times to calculate the mean and standard deviation.

### 4.8. Statistical Analysis

To investigate the agricultural traits of the 120 CNDH population, 125 plants were transplanted per each population, and 10 plants were randomly selected among them. All experimental data were replicated three times. For statistical analysis, SPSS statistical software (IMMSPSS Statistics, version 22, IBMSPSS Statistics, version 22, Redmond, WC, USA) was used, and after using one-way ANOVA, significant differences were calculated using Duncan’s Multiple Range Test (DMRT). In addition, R program ver3.6 (http://www.r-project.org/ (accessed on 30 November 2020)) was used to analyze the correlation coefficient of the investigated agricultural traits. The ‘corrplot’ package was used to construct the heatmap, and DMRT analyzed significant differences at *p* < 0.05 level.

## 5. Conclusions

Long awns played an important role in generation reproduction due to seed dispersion of wild rice, but the importance of awns in presently cultivated rice has disappeared. In particular, the existence of awn existed in a long, strong, and distinct shape in ancient rice, but as the times changed and human needs changed, the length of awn gradually became shorter or disappeared. We report here the detection of *OsDRPq3* at a major quantitative trait locus involved in awn development. *OsDRPq3* encodes a drooping leaf protein that regulates cell division. As the population in which the awn was developed, the yield was less, and the awn development was negatively adjusted to the yield. In addition, *OsDRPq3* is expressed at an early stage of the panicle formation stage and is implicated in awn development. It is suggested that awn loss is strongly favored and selected by breeders for yield increase because awn development causes yield loss during breeding from wild rice to cultivated rice. *OsDRPq3* and QTL regions can be used as a basis for the study of new molecular mechanisms for awn development. This study proposed that they can be used to create a breeding tree diagram of awns removed during rice domestication.

## Data Availability

The data presented in this study are available on request from the corresponding author.

## Supplementary Materials

The following are available online at https://www.mdpi.com/article/10.3390/plants11070851/s1. Table S1: The awn length, awn frequency per panicle, number of seeds per panicle, panicle length, yield from the 122 CNDH populations. Table S2: The correlations of awn length, awn frequency per panicle, number of seeds per panicle, panicle length, yield from the 122 CNDH populations. Table S3: QTL related to the agronomic characters of the Cheongcheong/Nagdong DH population. Table S4: Twenty-seven associate genes and their ORFs, which include various proteins related to awn development. Table S5: Primer list used for analysis of awn development-associated gene using qRT-PCR.
