# Supplementary material for: Phenotype and Genotype Interaction Underlying Distributive Characteristic for Awn Development in Rice
Source: Plants (Basel). 2022 Mar 23;11(7):851. doi: 10.3390/plants11070851 (PMC9002577; doi:10.3390/plants11070851)
Supplement: Supplementary file 1 [file plants-11-00851-s001.zip › plants-1642839-supplementary.pdf]

Table S1. The awn length, awn frequency per panicle, number of seeds per panicle, panicle length, yield from the 122 CNDH populations.

Plant trait	Years	Parents		DH population
		Cheongcheong	Nagdong	
Awn length (mm)	2019	0.0 ± 0.0	0.0 ± 0.0	11.1 ± 15.8
	2020	0.0 ± 0.0	0.0 ± 0.0	11.4 ± 16.1
Awn frequency per panicle (%)	2019	0.0 ± 0.0	0.0 ± 0.0	15.3 ± 25.5
	2020	0.0 ± 0.0	0.0 ± 0.0	15.8 ± 26.0
Number of seeds per panicle	2019	106.0 ± 1.4	123.0 ± 2.8	96.6 ± 25.5
	2020	111.5 ± 2.1	130.0 ± 1.4	99.1 ± 27.0
Panicle length (cm)	2019	20.6 ± 1.1	23.1 ± 1.0	18.8 ± 2.4
	2020	22.9 ± 0.9	25.8 ± 0.4	19.6 ± 3.7
Yield (kg/10a)	2019	350.9 ± 1.5	386.7 ± 1.9	374.1 ± 144.2
	2020	354.7 ± 4.9	394.5 ± 5.5	375.0 ± 144.5

[a] The data are presented in mean ± standard deviation.

Table S2. The correlations of awn length, awn frequency per panicle, number of seeds per panicle, panicle length, yield from the 122 CNDH populations.

Plant trait	Year	2019 Awn length (mm)	2020 Awn length (mm)	2019 Awn frequency per panicle (%)	2020 Awn frequency per panicle (%)	2019 Number of seeds per panicle	2020 Number of seeds per panicle	2019 Panicle length (cm)	2020 Panicle length (cm)	2019 Yield (kg/10a)	2020 Yield (kg/10a)
Awn length (mm)	2019	1.000									
	2020	0.997**	1.000								
Awn frequency per panicle (%)	2019	0.846**	0.844**	1.000							
	2020	0.850**	0.849**	0.997**	1.000						
Number of seeds per panicle	2019	-0.346**	-0.348**	-0.303**	-0.315**	1.000					
	2020	-0.331**	-0.336**	-0.305**	-0.317**	0.979**	1.000				
Panicle length (cm)	2019	-0.112	-0.124	-0.094	-0.120	0.478**	0.470**	1.000			
	2020	-0.067	-0.075	-0.059	-0.073	0.385**	0.426**	0.694**	1.000		
Yield (kg/10a)	2019	-0.364**	-0.359**	-0.402**	-0.411**	0.865**	0.852**	0.468**	0.378**	1.000	
	2020	-0.364**	-0.359**	-0.402**	-0.411**	0.865**	0.855**	0.468**	0.388**	0.999**	1.000

** significant at 0.01 level.

Table S3. QTL related to the agronomic characters of the Cheongcheong/Nagdong DH population.

Characteristics	Year	QTL	Chr.	Interval Marker	LOD	Additive effect	R^2	Increasing Effects
Awn length (mm)	2019	qAL6	6	RM3343-RM439	3.3	4.9	0.1	Cheongcheong
	2020	qAL6-1	6	RM3343-RM439	3.0	5.1	0.1	Cheongcheong
Panicle length (cm)	2019	qPL3	3	RM14330-RM218	3.2	0.9	0.1	Cheongcheong
		qPL7	7	RM20967-RM248	2.9	0.8	0.1	Cheongcheong
		qPL8	8	RM22861-RM22334	5.1	-0.8	0.1	Nagdong
	2020	qPL3-1	3	RM14330-RM218	3.6	1.2	0.2	Cheongcheong
Number of seeds per panicle	2019	qNS4	4	RM280-RM6909	2.7	-7.8	0.1	Nagdong
	2020	qNS4-1	4	RM280-RM6909	2.8	-8.0	0.1	Nagdong
		qNS12	12	RM27778-RM27442	2.7	-11.9	0.2	Nagdong
Awn frequency per panicle (%)	2019	qRA2	2	RM12662-RM6639	4.8	11.8	0.1	Cheongcheong
		qRA6	6	RM3343-RM439	3.5	8.3	0.1	Cheongcheong
		qRA9	9	RM3769-RM444	3.3	4.8	0.1	Cheongcheong
	2020	qRA2-1	2	RM12662-RM6639	4.6	-11.6	0.1	Nagdong
		qRA6-1	6	RM3343-RM439	3.5	4.8	0.1	Cheongcheong
		qRA9-1	9	RM3769-RM444	3.4	8.7	0.1	Cheongcheong
Yield (kg/10a)	2019	qYD8	8	RM506-RM1235	3.1	13.4	0.1	Cheongcheong
	2020	qYD8-1	8	RM506-RM1235	3.1	14.1	0.1	Cheongcheong

AL, awn length; PL, panicle length; NS, number of seed per panicle; RA, Awn frequency per panicle; YD, yield; [z] Interval markers are those within the significance threshold on each border of the QTL rang; [y] The proportion of evaluated phenotype variations attributable to a particular QTL was estimated using the coefficient of determination (R^2); [x] Positive values of the additive effect indicate that alleles from Samgang are in the direction of increases in the traits; [w] Increased allele is the source of the allele causing an increase in the measured trait.

Table S4. Twenty-seven candidate genes and their ORFs, which include various proteins related to awn development.

Locus	Description
LOC_Os02g08220	Helix-loop-helix DNA-binding domain containing protein.
LOC_Os04g47810	Helix-loop-helix DNA-binding domain containing protein.
LOC_Os08g08160	Helix-loop-helix DNA-binding domain containing protein.
LOC_Os08g16030	Helix-loop-helix DNA-binding domain containing protein.
LOC_Os08g31950	Helix-loop-helix DNA-binding domain containing protein
LOC_Os08g40230	Helix-hairpin-helix motif, class 2 domain containing protein.
LOC_Os04g53990	Basic helix-loop-helix dimerisation region bHLH domain containing protein.
LOC_Os04g51070	Basic helix-loop-helix dimerisation region bHLH domain containing protein.
LOC_Os03g15440	Basic helix-loop-helix dimerisation region bHLH domain containing protein.
LOC_Os03g07540	Basic helix-loop-helix dimerisation region bHLH domain containing protein.
LOC_Os03g08930	Basic helix-loop-helix dimerisation region bHLH domain containing protein.
LOC_Os08g37290	Basic helix-loop-helix dimerisation region bHLH domain containing protein.
LOC_Os08g37730	Basic helix-loop-helix dimerisation region bHLH domain containing protein.
LOC_Os08g38210	Basic helix-loop-helix dimerisation region bHLH domain containing protein.
LOC_Os08g39630	Basic helix-loop-helix dimerisation region bHLH domain containing protein.
LOC_Os12g06330	Basic helix-loop-helix dimerisation region bHLH domain containing protein.
LOC_Os04g52770	Similar to Long hypocotyl in far-red 1 (bHLH-like protein HFR1) (Basic helix-loop-helix FBI1 protein)
LOC_Os07g08440	Similar to Phytochrome-interacting factor 3 (Phytochrome-associated protein 3) (Basic helix-loop-helix protein 8)
LOC_Os12g10140	Winged helix repressor DNA-binding domain containing protein.

LOC_Os08g35310	Winged helix repressor DNA-binding domain containing protein.
LOC_Os08g19420	Winged helix repressor DNA-binding domain containing protein.
LOC_Os08g07260	Winged helix repressor DNA-binding domain containing protein.
LOC_Os03g11600	Drooping leaf protein.
LOC_Os03g11680	Similar to Zinc-finger protein KNUCKLES.
LOC_Os04g52190	Similar to Vacuolar sorting receptor 7 precursor (AtVSR7) (Epidermal growth factor receptor-like protein 3)
LOC_Os07g06620	YABBY protein (OsYAB1) (Filamentous flower protein 1).
LOC_Os08g40900	Similar to Auxin response factor 5 (Transcription factor MONOPTEROS)

Table S5. Primer list used for analysis of awn development associated gene using qRT-PCR.

Gene	Forward/Reverse	Sequence (5'-3')
OsDRPq3	Forward	GCCTCCCATCTGCTTACAAC
	Reverse	GTGAGGGATATCTGGCTTGG
OsActin	Forward	TGCTGATCGTATGAGCAAGG
	Reverse	TCTTTCTGGTGGTGCAATCA
LOC_Os08g16030	Forward	GCACCGTGCTTCTCAACC
	Reverse	AAGCGTGGCCGCTTGTAG
LOC_Os08g37290	Forward	GGATTTCTTGAGCCAGATGC
	Reverse	CTGCAATTTTGGTGGTCTGA
LOC_Os04g52770	Forward	CAGTGGGAGCAAACAACAGA
	Reverse	CTTCCATCAGCTTCGCTACC
LOC_Os07g08440	Forward	GCCTCCCATCTGCTTACAAC
	Reverse	GTGAGGGATATCTGGCTTGG
LOC_Os12g10140	Forward	TTGTACGCGCAAGAACTCAT
	Reverse	ATCGAGAAGACCAAGCTGGA
LOC_Os02g08220	Forward	GCGTTCAAGAAACCGAGACT
	Reverse	CCTGTCCCCTAGCTTCTCCT
LOC_Os07g06620	Forward	GTACCCAATGCCAACCAGTC
	Reverse	GATGGGACACGCTGTCTTTT
